# Supplementary material for: Reforming the registration policy of female sex workers in Senegal? Evidence from a discrete choice experiment
Source: PLoS One. 2023 Aug 16;18(8):e0289882. doi: 10.1371/journal.pone.0289882 (PMC10431633; doi:10.1371/journal.pone.0289882)
Supplement: S1 Table — The findings from the focus groups regarding the favoured interventions to improve the current registration policy among non-registered FSWs. (DOCX) [file pone.0289882.s001.docx]

**S1 Table. Favoured interventions to improve the current registration policy among non-registered FSWs.**

|  | Group 1 | Group 2 |
| --- | --- | --- |
| 1 | Changing the design of the booklet (smaller/removing the word 'health booklet'/no writing on it apart from the name) | Access to mental health advice or support |
| 2 | Access to literacy, training or skills development for FSWs | Quality medical care (friendly health staff, good medical care) |
| 3 | Removal of the central police database | Integration of medical visits, i.e. they can make the visit on any day |
