# Supplementary material for: Reforming the registration policy of female sex workers in Senegal? Evidence from a discrete choice experiment
Source: PLoS One. 2023 Aug 16;18(8):e0289882. doi: 10.1371/journal.pone.0289882 (PMC10431633; doi:10.1371/journal.pone.0289882)
Supplement: S2 Table — (DOCX) [file pone.0289882.s002.docx]

**S2 Table. Focus group methods and study sample sizes.**

| **Wave** | **Method** | **Registered FSW** | **Non-registered FSW** | **Total** |
| --- | --- | --- | --- | --- |
| Wave 2 (2017) | Quantitative survey | 255 | 257 | 512 |
| Wave 3 (2020) | Focus groups | 2 | 2 | 4 |
| Wave 3 (2020) | Discrete choice experiment | 241 | 273 | 514 |
