# Supplementary material for: Reforming the registration policy of female sex workers in Senegal? Evidence from a discrete choice experiment
Source: PLoS One. 2023 Aug 16;18(8):e0289882. doi: 10.1371/journal.pone.0289882 (PMC10431633; doi:10.1371/journal.pone.0289882)
Supplement: S1 File — The script used by enumerators to present the DCE to participants. (DOCX) [file pone.0289882.s003.docx]

**S1 File. DCE manuscript.** The script used by enumerators to present the DCE to participants.

Sex work is legal and regulated in Senegal. To practise it legally, you must:

- register with the authorities
- undergo routine monthly medical check-ups and test negative for STIs, at a monthly cost of around FCFA 2,000
- carry an up-to-date health booklet, confirming your compliance with routine medical check-ups

Your ID and photo will also be kept at the hospital and in a police file to prove your registration. These records will not be deleted if you leave sex work.

Being registered will not affect your human rights, you and your family members will still be able to travel outside Senegal, have access to the judicial system or the profession of your choice.

Senegalese law stipulates that unregistered sex workers risk a fine of 30,000 FCFA and a prison sentence of up to 6 months if arrested by the police.

We will now describe the different variants of the registration policy. There are ten questions, and each question presents two policy options that we would like you to compare. We'll ask you to tell us whether you prefer policy A, or policy B or neither because you prefer your current situation.

Each policy option described depends on the following elements: file, proof of registration, confidentiality at the hospital, free medical visits, and psychosocial follow-up.

Remember, there are no right or wrong answers.

Once the task has been explained, the enumerator shows the participant the policy options. Whilst showing the participant the policy options on the screen, the enumerator asks:

1. Imagine two registration policies: Policy A and policy B. Which do you prefer?

The responses to the first question are Policy A, Policy B or neither (opt-out) e.g., prefer their current situation. If the participant choses to opt-out, a question that forces a choice between policy A or B follows:

1. Do you prefer Policy A or Policy B?
