# Supplementary material for: Reforming the registration policy of female sex workers in Senegal? Evidence from a discrete choice experiment
Source: PLoS One. 2023 Aug 16;18(8):e0289882. doi: 10.1371/journal.pone.0289882 (PMC10431633; doi:10.1371/journal.pone.0289882)
Supplement: Attachment — Submitted filename: PONE_comments2.docx [file pone.0289882.s005.docx]

PONE-D-22-30805

Reforming the registration policy of Female Sex Workers in Senegal? Evidence from a discrete choice experiment

Thank you very much for your invitation to revise and resubmit our paper “Reforming the registration policy of Female Sex Workers in Senegal? Evidence from a discrete choice experiment”. We very much appreciate the time taken by referees to review our responses to their comments. We have carefully gone through the list of comments and suggestions in order to improve the paper. We hope that it is now acceptable for publication. In the rest of this letter, we cover the changes that we have made in response to the suggestions, first repeating the comment or question from each referee and then reporting the modified paragraph in the indented text in the changes are not too important. Within our responses, we also include the relevant section of revised text, if appropriate.

We would also like to confirm that this project received funding from MRC Public Health Intervention Development Scheme (MR/T00262X/1) and the funders had no role in study design, data collection and analysis, decision to publish, or preparation of the manuscript. We would also like to confirm that the authors have declared that no competing interests exist. In addition, the data has been made available at the UCL data depositary. Thank you in advance for updating the Data Availability statement on our behalf.

When submitting your revision, we need you to address these additional requirements.

1. Please ensure that your manuscript meets PLOS ONE's style requirements, including those for file naming. The PLOS ONE style templates can be found at

[https://journals.plos.org/plosone/s/file?id=wjVg/PLOSOne_formatting_sample_main_body.pdf](https://eur01.safelinks.protection.outlook.com/?url=https%3A%2F%2Fjournals.plos.org%2Fplosone%2Fs%2Ffile%3Fid%3DwjVg%2FPLOSOne_formatting_sample_main_body.pdf&data=05%7C01%7Cs.szawlowski%40ucl.ac.uk%7Cd79e90fe4f444dc3b1f508db44dfc7dc%7C1faf88fea9984c5b93c9210a11d9a5c2%7C0%7C0%7C638179500532714059%7CUnknown%7CTWFpbGZsb3d8eyJWIjoiMC4wLjAwMDAiLCJQIjoiV2luMzIiLCJBTiI6Ik1haWwiLCJXVCI6Mn0%3D%7C3000%7C%7C%7C&sdata=02yKWwBpYepMDMXyM6iuXuR6SxxMfKqrjSW%2FVfcn9hg%3D&reserved=0) and

[https://journals.plos.org/plosone/s/file?id=ba62/PLOSOne_formatting_sample_title_authors_affiliations.pdf](https://eur01.safelinks.protection.outlook.com/?url=https%3A%2F%2Fjournals.plos.org%2Fplosone%2Fs%2Ffile%3Fid%3Dba62%2FPLOSOne_formatting_sample_title_authors_affiliations.pdf&data=05%7C01%7Cs.szawlowski%40ucl.ac.uk%7Cd79e90fe4f444dc3b1f508db44dfc7dc%7C1faf88fea9984c5b93c9210a11d9a5c2%7C0%7C0%7C638179500532714059%7CUnknown%7CTWFpbGZsb3d8eyJWIjoiMC4wLjAwMDAiLCJQIjoiV2luMzIiLCJBTiI6Ik1haWwiLCJXVCI6Mn0%3D%7C3000%7C%7C%7C&sdata=bZMS9274tB4ZggzC9xGu0UG0qQUuK84qvv5DyHKQ55U%3D&reserved=0).

We have now amended our manuscript to ensure that all PLOS ONE’s style requirements are met.

2. Please include a complete copy of PLOS’ questionnaire on inclusivity in global research in your revised manuscript. Our policy for research in this area aims to improve transparency in the reporting of research performed outside of researchers’ own country or community. The policy applies to researchers who have travelled to a different country to conduct research, research with Indigenous populations or their lands, and research on cultural artefacts. The questionnaire can also be requested at the journal’s discretion for any other submissions, even if these conditions are not met.  Please find more information on the policy and a link to download a blank copy of the questionnaire here: [https://journals.plos.org/plosone/s/best-practices-in-research-reporting](https://eur01.safelinks.protection.outlook.com/?url=https%3A%2F%2Fjournals.plos.org%2Fplosone%2Fs%2Fbest-practices-in-research-reporting&data=05%7C01%7Cs.szawlowski%40ucl.ac.uk%7Cd79e90fe4f444dc3b1f508db44dfc7dc%7C1faf88fea9984c5b93c9210a11d9a5c2%7C0%7C0%7C638179500532714059%7CUnknown%7CTWFpbGZsb3d8eyJWIjoiMC4wLjAwMDAiLCJQIjoiV2luMzIiLCJBTiI6Ik1haWwiLCJXVCI6Mn0%3D%7C3000%7C%7C%7C&sdata=3DghhzYOypVovLBb4TRyHffZ90HD0rCvvExBx8Rfb74%3D&reserved=0). Please upload a completed version of your questionnaire as Supporting Information when you resubmit your manuscript.

We have included the questionnaire.

3. Please provide additional details regarding participant consent. In the ethics statement in the Methods and online submission information, please ensure that you have specified what type you obtained (for instance, written or verbal, and if verbal, how it was documented and witnessed). If your study included minors, state whether you obtained consent from parents or guardians. If the need for consent was waived by the ethics committee, please include this information.

Thank you for your comment. We have now added the following text:

Recruitment, Page 6, paragraph 1: “Only participants 18 years old and over and living in Dakar were invited to take part in the study, no minors were included.”

Survey, Page 6, paragraph 2: “For all waves, if interested in participating, potential participants were invited to come to one of the four named sites and informed consent followed the same process. Consent was in-person and written. Before consenting to take part in the study, the research purpose, processes and what was expected of them were explained to them. In addition, potential participants were made aware of data management procedures and that all personal information collected during the study will be de-identified to ensure its confidentiality. Respondents were informed that they could withdraw from the study at any time. When ready to consent, all respondents signed a form agreeing to take part in the study.”

4. We note that the grant information you provided in the ‘Funding Information’ and ‘Financial Disclosure’ sections do not match.

When you resubmit, please ensure that you provide the correct grant numbers for the awards you received for your study in the ‘Funding Information’ section.

We have now corrected this to MRC Public Health Intervention Development Scheme (MR/T00262X/1)

5. Thank you for stating the following financial disclosure:

“No”

At this time, please address the following queries:

a) Please clarify the sources of funding (financial or material support) for your study. List the grants or organizations that supported your study, including funding received from your institution.

The study was funding by the Medical Research Council, a UK research council under the competitive scheme Public Health Intervention Development Scheme

State what role the funders took in the study. If the funders had no role in your study, please state: “The funders had no role in study design, data collection and analysis, decision to publish, or preparation of the manuscript.”

Please note: The funders had no role in study design, data collection and analysis, decision to publish, or preparation of the manuscript. We have mentioned this in our cover letter.

b) If any authors received a salary from any of your funders, please state which authors and which funders.

No salary was received from the funder.

d) If you did not receive any funding for this study, please state: “The authors received no specific funding for this work.”

Please note: The authors received no specific funding for this work. We have included this in our cover letter.

Please include your amended statements within your cover letter; we will change the online submission form on your behalf.

Thank you for amending the online submission on our behalf. We have included the amended statements in the cover letter.

6. Thank you for stating the following in your Competing Interests section:

“NO authors have competing interests”

Please complete your Competing Interests on the online submission form to state any Competing Interests. If you have no competing interests, please state ""The authors have declared that no competing interests exist."", as detailed online in our guide for authors at [http://journals.plos.org/plosone/s/submit-now](https://eur01.safelinks.protection.outlook.com/?url=http%3A%2F%2Fjournals.plos.org%2Fplosone%2Fs%2Fsubmit-now&data=05%7C01%7Cs.szawlowski%40ucl.ac.uk%7Cd79e90fe4f444dc3b1f508db44dfc7dc%7C1faf88fea9984c5b93c9210a11d9a5c2%7C0%7C0%7C638179500532714059%7CUnknown%7CTWFpbGZsb3d8eyJWIjoiMC4wLjAwMDAiLCJQIjoiV2luMzIiLCJBTiI6Ik1haWwiLCJXVCI6Mn0%3D%7C3000%7C%7C%7C&sdata=fuLElmLYCkhotFiTMxCMzsssf%2BPge2Qsq%2FfHKcdr18c%3D&reserved=0)

This information should be included in your cover letter; we will change the online submission form on your behalf.

We have completed the online form.

7. In your Data Availability statement, you have not specified where the minimal data set underlying the results described in your manuscript can be found. PLOS defines a study's minimal data set as the underlying data used to reach the conclusions drawn in the manuscript and any additional data required to replicate the reported study findings in their entirety. All PLOS journals require that the minimal data set be made fully available. For more information about our data policy, please see [http://journals.plos.org/plosone/s/data-availability](https://eur01.safelinks.protection.outlook.com/?url=http%3A%2F%2Fjournals.plos.org%2Fplosone%2Fs%2Fdata-availability&data=05%7C01%7Cs.szawlowski%40ucl.ac.uk%7Cd79e90fe4f444dc3b1f508db44dfc7dc%7C1faf88fea9984c5b93c9210a11d9a5c2%7C0%7C0%7C638179500532714059%7CUnknown%7CTWFpbGZsb3d8eyJWIjoiMC4wLjAwMDAiLCJQIjoiV2luMzIiLCJBTiI6Ik1haWwiLCJXVCI6Mn0%3D%7C3000%7C%7C%7C&sdata=A%2BHtZQpxcPOy7GMO4qXM602R8nDRz8Ap9WrAwTK2sCU%3D&reserved=0).

""Upon re-submitting your revised manuscript, please upload your study’s minimal underlying data set as either Supporting Information files or to a stable, public repository and include the relevant URLs, DOIs, or accession numbers within your revised cover letter. For a list of acceptable repositories, please see [http://journals.plos.org/plosone/s/data-availability#loc-recommended-repositories](https://eur01.safelinks.protection.outlook.com/?url=http%3A%2F%2Fjournals.plos.org%2Fplosone%2Fs%2Fdata-availability%23loc-recommended-repositories&data=05%7C01%7Cs.szawlowski%40ucl.ac.uk%7Cd79e90fe4f444dc3b1f508db44dfc7dc%7C1faf88fea9984c5b93c9210a11d9a5c2%7C0%7C0%7C638179500532714059%7CUnknown%7CTWFpbGZsb3d8eyJWIjoiMC4wLjAwMDAiLCJQIjoiV2luMzIiLCJBTiI6Ik1haWwiLCJXVCI6Mn0%3D%7C3000%7C%7C%7C&sdata=zv%2B8fHYEJFB0sv92Q8rnt26vZg%2FNdJYLTxPT%2BdF285Q%3D&reserved=0). Any potentially identifying patient information must be fully anonymized.

Important: If there are ethical or legal restrictions to sharing your data publicly, please explain these restrictions in detail. Please see our guidelines for more information on what we consider unacceptable restrictions to publicly sharing data: [http://journals.plos.org/plosone/s/data-availability#loc-unacceptable-data-access-restrictions](https://eur01.safelinks.protection.outlook.com/?url=http%3A%2F%2Fjournals.plos.org%2Fplosone%2Fs%2Fdata-availability%23loc-unacceptable-data-access-restrictions&data=05%7C01%7Cs.szawlowski%40ucl.ac.uk%7Cd79e90fe4f444dc3b1f508db44dfc7dc%7C1faf88fea9984c5b93c9210a11d9a5c2%7C0%7C0%7C638179500532714059%7CUnknown%7CTWFpbGZsb3d8eyJWIjoiMC4wLjAwMDAiLCJQIjoiV2luMzIiLCJBTiI6Ik1haWwiLCJXVCI6Mn0%3D%7C3000%7C%7C%7C&sdata=IPM24V1wZGhwGVkh1raFVrZiHZz9hsBCpZ5sE8lfyHk%3D&reserved=0). Note that it is not acceptable for the authors to be the sole named individuals responsible for ensuring data access.

We will update your Data Availability statement to reflect the information you provide in your cover letter.

We will submit this as a supporting information file.

8. We note that you have indicated that data from this study are available upon request. PLOS only allows data to be available upon request if there are legal or ethical restrictions on sharing data publicly. For more information on unacceptable data access restrictions, please see [http://journals.plos.org/plosone/s/data-availability#loc-unacceptable-data-access-restrictions](https://eur01.safelinks.protection.outlook.com/?url=http%3A%2F%2Fjournals.plos.org%2Fplosone%2Fs%2Fdata-availability%23loc-unacceptable-data-access-restrictions&data=05%7C01%7Cs.szawlowski%40ucl.ac.uk%7Cd79e90fe4f444dc3b1f508db44dfc7dc%7C1faf88fea9984c5b93c9210a11d9a5c2%7C0%7C0%7C638179500532714059%7CUnknown%7CTWFpbGZsb3d8eyJWIjoiMC4wLjAwMDAiLCJQIjoiV2luMzIiLCJBTiI6Ik1haWwiLCJXVCI6Mn0%3D%7C3000%7C%7C%7C&sdata=IPM24V1wZGhwGVkh1raFVrZiHZz9hsBCpZ5sE8lfyHk%3D&reserved=0).

In your revised cover letter, please address the following prompts:

a) If there are ethical or legal restrictions on sharing a de-identified data set, please explain them in detail (e.g., data contain potentially sensitive information, data are owned by a third-party organization, etc.) and who has imposed them (e.g., an ethics committee). Please also provide contact information for a data access committee, ethics committee, or other institutional body to which data requests may be sent.

b) If there are no restrictions, please upload the minimal anonymized data set necessary to replicate your study findings as either Supporting Information files or to a stable, public repository and provide us with the relevant URLs, DOIs, or accession numbers. For a list of acceptable repositories, please see [http://journals.plos.org/plosone/s/data-availability#loc-recommended-repositories](https://eur01.safelinks.protection.outlook.com/?url=http%3A%2F%2Fjournals.plos.org%2Fplosone%2Fs%2Fdata-availability%23loc-recommended-repositories&data=05%7C01%7Cs.szawlowski%40ucl.ac.uk%7Cd79e90fe4f444dc3b1f508db44dfc7dc%7C1faf88fea9984c5b93c9210a11d9a5c2%7C0%7C0%7C638179500532714059%7CUnknown%7CTWFpbGZsb3d8eyJWIjoiMC4wLjAwMDAiLCJQIjoiV2luMzIiLCJBTiI6Ik1haWwiLCJXVCI6Mn0%3D%7C3000%7C%7C%7C&sdata=zv%2B8fHYEJFB0sv92Q8rnt26vZg%2FNdJYLTxPT%2BdF285Q%3D&reserved=0).

We will update your Data Availability statement on your behalf to reflect the information you provide.

There are no restrictions so we will upload the data.

9. PLOS requires an ORCID iD for the corresponding author in Editorial Manager on papers submitted after December 6th, 2016. APlease ensure that you have an ORCID iD and that it is validated in Editorial Manager. To do this, go to ‘Update my Information’ (in the upper left-hand corner of the main menu), and click on the Fetch/Validate link next to the ORCID field. This will take you to the ORCID site and allow you to create a new iD or authenticate a pre-existing iD in Editorial Manager. Please see the following video for instructions on linking an ORCID iD to your Editorial Manager account: [https://www.youtube.com/watch?v=_xcclfuvtxQ](https://eur01.safelinks.protection.outlook.com/?url=https%3A%2F%2Fwww.youtube.com%2Fwatch%3Fv%3D_xcclfuvtxQ&data=05%7C01%7Cs.szawlowski%40ucl.ac.uk%7Cd79e90fe4f444dc3b1f508db44dfc7dc%7C1faf88fea9984c5b93c9210a11d9a5c2%7C0%7C0%7C638179500532714059%7CUnknown%7CTWFpbGZsb3d8eyJWIjoiMC4wLjAwMDAiLCJQIjoiV2luMzIiLCJBTiI6Ik1haWwiLCJXVCI6Mn0%3D%7C3000%7C%7C%7C&sdata=wGRj25xExU73CT255Ryce6L1IPhEzKOqRRxP6l9tBtw%3D&reserved=0).

 Aurelia, you need to edit this on your actual account.

**Comments to the Author**

**Reviewer #1**

 ABSTRACT
• The second and third sentence of the abstract seem redundant. Simplify this to include more in the background of the abstract.
• Abstract could use further description of the methods and results of the discrete choice experiment
•Include quantitative results in the abstract
• Use past tense

We have now rewritten the abstract following your recommendations:

Abstract, page 2

“Abstract: Evidence suggests that treating sexually transmitted infections (STIs) amongst female sex workers (FSWs) is a cost-effective strategy to reduce the spread of HIV/AIDS. Senegal is the only African country where sex work is regulated by a public health policy which aims to monitor and routinely treat STIs. The law requires FSWs to be at least 21 years old, register with a health centre and the police, carry an up-to-date registration booklet, attend monthly health check-ups, and test negative for STIs. Despite health and legal benefits of registration, 80% of FSWs in Senegal are not registered. Hence, the potential health benefits of the policy have not materialised. To understand why FSWs do not want to register and to define policy changes that would increase the registration rate of FSWs in Senegal, we designed and implemented a discrete choice experiment (DCE) completed by 241 registered and 273 non-registered FSWs. Participants made choices between a series of hypothetical but realistic registration policy changes. Conditional logit models were used to analyse the DCE data. The results highlighted that confidentiality at the health facility was an important element, registered and non-registered FWs were respectively 26.0 percentage points (pp) and 22.1 pp more likely to prefer a policy that guaranteed confidentiality at the health centre. Similarly, both groups preferred a policy where their health record was only held at the health centre and not with the police. Several interventions to increase FSW registration rate and improve their wellbeing may be implemented without modifying the law. For example, the introduction of psychosocial support in the registration policy package, replacing the registration booklet by a QR code, the use of electronic medical files and the integration of FSWs routine visits with maternal health appointments to increase confidentiality have the potential to encourage registration of FSWs.”

INTRODUCTION
• There are some important readability concerns in this section, including incomplete/run-on sentences (see paragraph 2). Please review carefully and correct these issues.

We have rewritten and proofread this section as follows:

Introduction, page 3, paragraph 2.

“The registration policy originates in old French colonial laws [6]. In the second half of the 19th century, the devastating consequences of syphilis, especially in French colonial towns and among troops, was a real concern for the colonial authorities. They found that the hospitalization of soldiers compromised the performance of the military. At the time, medical authorities determined that sex work was at the origin of syphilis transmission. To respond, French legislation introduced regulation of sex work to prevent the spread of STIs in colonies. In 1849, a decree introduced compulsory medical visits for sex workers and required them to register with the health authorities and carry a health booklet [7, 8]. Sex work was outlawed by the colonial authorities in 1946 and many Francophone countries dropped regulation of sex work after independence. However, Senegal did not. The Senegalese Minister of the Interior at the time decided that regulating sex work was safer than outlawing it to limit its expansion [9] . As a result, since February 1966, Senegalese FSWs older than 21 years old have been required to register with a health centre and attend routine health visits to be tested and treated for STIs [10]. Upon registration, a registration file is created at the health centre and shared with the police. In addition, an official registration booklet is issued (called “*carnet sanitaire*”) which keeps a record of the visits made to the appointed health centre. If FSWs test positive for any STI, apart from HIV, the booklet is kept at the health centre during the whole course of treatment. Screened HIV-positive FSWs are referred to higher levels of care, and adherence to antiretroviral treatment is monitored during routine visits. FSWs who are arrested and fail to present an up-to-date registration booklet (either because they are not registered, do not comply with routine visits, or are currently being treated for an STI) may incur a prison sentence of between 2 and 6 months (Code pénal articles 319/ 325, law n° 66-21). Legally, when a FSW chooses to leave sex work she must notify both the health centre and the police, and the registration file held by both authorities should be destroyed. However, in actuality, due to the common belief that FSWs are likely to return to sex work and the want to minimise administrative procedures, the destruction of records are not consistently practiced [10]. Since the introduction of the registration policy no significant change has been implemented, except for minor adjustments to the appearance of the registration booklet.”

• All of the references used are quite old, e.g. cost-effectiveness and importance of addressing the health needs of FSW. Please include more recent and additional citations/evidence to justify your statements.

We have now added the following references:

o There are a number of statements that do not include any reference at all but require them, including most of paragraph 2 and 3.

Thank you for your comments. We have now amended paragraph 2 and 3 to ensure that the relevant references are included.

• You mention that in West Africa, HIV infections are concentrated among FSW. What about other key populations? This statement could benefit from expounding that FSW are one key group that are disproportionately affected by HIV.

HIV epidemic in Senegal is concentrated among key populations and as a results MSMS and injected drug users are also disproportionally affected by HIV. We have now rewritten this sentence to reflect this:

Introduction, page 3, paragraph 1: “FSWs are one the key groups disproportionally affected with HIV”.

• I’m not sure what you mean when you say “For medical authorities sex work was at the origin of syphilis transmission.”

We have reworded this sentence:

Introduction, page 3, paragraph 2: “At the time, medical authorities determined that sex work was at the origin of syphilis transmission. To respond, French legislation introduced the regulation of sex work to prevent the spread of STIs in colonies.”

• It is unusual to include a detailed summary of the methods and results in the introduction. [https://journals.plos.org/plosone/s/submission-guidelines](https://eur01.safelinks.protection.outlook.com/?url=https%3A%2F%2Fjournals.plos.org%2Fplosone%2Fs%2Fsubmission-guidelines&data=05%7C01%7Cs.szawlowski%40ucl.ac.uk%7Cd79e90fe4f444dc3b1f508db44dfc7dc%7C1faf88fea9984c5b93c9210a11d9a5c2%7C0%7C0%7C638179500532714059%7CUnknown%7CTWFpbGZsb3d8eyJWIjoiMC4wLjAwMDAiLCJQIjoiV2luMzIiLCJBTiI6Ik1haWwiLCJXVCI6Mn0%3D%7C3000%7C%7C%7C&sdata=BL20GulfVbJ1UWCxvVmxQeXfpUZOvRe8Hqq%2BN4qoSi4%3D&reserved=0)

Thank you for your comment, this has been removed.

METHODS
• You mention that you tried to contact FSWs who had taken part in the 2015 and 2017 surveys, but of what? A larger study? An IBBS? A serial cross-sectional study? Please provide more context here

The text has been amended to further explain the previous cohort study:

Data collection and ethics, page 5: “The DCE was embedded within the third wave of data collection of a longitudinal cohort study of 600 FSWs in Dakar, Senegal, noted earlier, and was carried out from 29 June to 28 July 2020. Data was collected face-to-face by trained enumerators in private rooms located near four of out five of the STI health centres in Dakar, Senegal (Pikine, Rufisque, Mbao, and Sebikotane). These STI health centres are now referred to as the study sites.

The cohort study follows FSWs in Dakar, Senegal and it was conceived to document and understand the socio-economic and behavioural determinants of risky sexual behaviours among FSWs. The cohort study began in 2015 (wave 1) and wave 2 was conducted in 2017. The aim of the third wave of data collection in 2020 was to define a policy change to address the main barriers of registration, increase the benefits of registration, provide evidence on the feasibility and to evaluate the effectiveness and cost-effectiveness of this policy change. Each wave of data collection for the cohort study received appropriate individual ethical clearance. Wave 3 received approval from the National Ethics Committee in Senegal (SEN19/88 n00037MSAS/DGS/DLM/DLSI) and the University College London ethics committee (17341/001).”

• How was recruitment done? Was there any sampling process employed at the health-centres? Was there a specific process used by leaders of the FSW groups?

We have included a new section which discusses the recruitment further in the paper, as follows:

“Recruitment

For the first wave in 2015, registered FSWs were recruited by the midwife in charge of their compulsory monthly health check. All active registered FSWs from the four study sites were contacted to participate in the study. There was no sampling process employed at the health-centres. Non-registered FSWs were contacted and recruited by leaders of FSW groups. Again, there was no specific process used by leaders of the FSW groups. The total study sample included a similar proportion of registered and non-registered FSWs. All participants gave permission to be recontacted for future waves.

The same methodology was used to recruit participants in wave 2 (2017) and wave 3 (2020). For wave 2, all participants in wave 1 were contacted to participate. For wave 3, all participants that participated in wave 2 were contacted alongside those that participated in wave 1 but did not participate in wave 2. Participants were initially contacted via telephone and if not contactable, peer FSWs reached out to non-registered sex workers and midwives approached registered FSWs. For both wave 2 and 3, to maintain a cohort of 600 FSWs new registered were recruited at STI health centres and non-registered FSWs were recruited by leaders of FSWs groups. Only participants 18 years old and over and living in Dakar were invited to take part in the study, no minors were included.”

• It would be helpful to have more description on the different attributes and how they were determined for inclusion. You mention a literature review – what was reviewed? What were the results of that review that led you to these attributes?

Thank you for your comment. We made a mistake in noting that a literature review supported the choices of attributes to be included in the DCE. Please find below the updated text:

The attributes included in the DCE were based on findings from previous research conducted on the registration policy in Senegal (Ito et al, 2018) and from discussion with policy experts.

We then confronted those attributes to study participants using interviews and focus group discussions. Note this qualitative study is also under revision in PLOSOne (Procureur et al, 2022).

We are now providing this information in the method and rewrote the following section:

“Selection and development of attributes and levels for the DCE

The attributes included in the DCE were chosen using findings from previous research conducted on the registration policy in Senegal [11] and discussions with policy experts, government officials, FSW group leaders, and FSWs (registered and non-registered). Previous research found that FSWs have a low level of well-being due to the stigma attached to the current design of the registration policy [11] and the further discussions allowed the identification of key policy attributes in a FSWs registration decision which are feasible to change in legislation.

Focus groups with Female sex workers

In wave 3 of the data collection there were four focus groups, two with registered FSWs and two with non-registered FSW. Focus groups were conducted in a ‘non-directive’ approach, facilitated by a topic guide to understand their views on the registration policy and lasted on average 90 minutes. FSWs recruited for the focus group discussions had participated in at least one previous wave of the cohort study and were called at random. Four focus groups (two for registered and two for non-registered FSWs) were conducted with six FSWs in each focus group. The focus groups founded that nine out of 11 (81.8%) non-registered noted that they did not want their profession to be discovered by their loved ones. The findings from the focus groups regarding the favoured interventions to improve the current registration policy among non-registered FSWs along with the methods used and sample size can be found in the S1 File.

DCE attributes and levels

The attributes covered key policy attributes of the registration policy and are presented in Fig 1 (column 1)). The chosen attributes include the registration file, the design of the registration proof, confidentiality of routine visits, costs of routine visits and psychological support.

The first attribute shown in Fig 1 is the location, or with which authority, a FSWs file is held and for how long their file remains active. According to the law, at the time of registration at the health centre a file is created. This file is then shared with the police. The lawful process for when a FSW decides to leave sex work is that she should notify the health centre and then both the health centre and the police should destroy her file. However, in practice, the location of where and the length of time that the file is held for is often not in keeping with regulation. Files collated at the health centre are not always shared with the police and because of the perceived likelihood that they will return to sex work, health centres do not consistently destroy files when a FSW notifies them that they have left sex work. Discussions with FSW group leaders and FSWs highlighted that having sex work files held by police for life may cause difficulties due to stigma by the police.

The second attribute was the design of the registration proof. To work legally, registered FSWs must carry an up-to-date registration booklet whilst soliciting clients which when not working, they must hide at home. The current booklet easily identifies an individual as a FSW and poses an increased risk of others discovering her sex work activities. As noted, previous research using earlier waves of data found that the booklet was one of the main reasons for non-registration [11]. To respond to this finding, the Ministry of Health in Senegal have attempted changed the appearance of the registration booklet to make it more discreet and less stigmatizing, however there has been no impact on the registration rate [11].

Thirdly, confidentiality of routine visits, was highlighted as an important attribute. Compulsory health visits, outlined in law, for FSWs are held on a specific day (Thursday) which the general population are aware of [5]. FSWs are often forced into attending health centres in their local area due to financial and time constraints. Attending health centre visits locally increases the likelihood of a FSW seeing someone they know and having their sex work activities discovered by others. The focus groups found that 16% of non-registered FSWs believe that medical visits would not be confidential, whereas 98% of FSWs who are registered declare that confidentially is guaranteed (See S1 File). This attribute captures any perceived or real risks of having the sex worker status disclosed whilst at the hospital, either because the hospital staff reveals that the patient is a FSW or if the services for FSWs are not integrated and the FSWs meets someone she knows while queuing or receiving the medical services.

The cost of visit was included as the fourth attribute in the DCE. Although not the most important reason for non-registration in the focus groups, some FSWs expressed that they were unable to meet this expense. FSWs must pay for the compulsory appointment, all STI tests and any treatment. On average, FSWs pay 2000 FCFA (2.63 GBP) per visit. The amount that a FSW pays per visit is not determined by law and varies from health centre to health centre.

The focus groups founded that psychological support was a preferred intervention to increase the likelihood of registration. This fifth attribute is especially important as findings suggest that registering reduces well-being [11].

For each attribute several levels were defined, these are presented in column (2). The levels were determined with the support of policy experts, government officials, FSW group leaders, and FSWs respectively to ensure the feasibility of policy change and suitability.”

• How were folks selected for the focus group discussions?

To cover this in the manuscript we have added an additional paragraph which reads:

“Focus groups with Female sex workers

In wave 3 of the data collection there were four focus groups, two with registered FSWs and two with non-registered FSW. Focus groups were conducted in a ‘non-directive’ approach, facilitated by a topic guide to understand their views on the registration policy and lasted on average 90 minutes. FSWs recruited for the focus group discussions had participated in at least one previous wave of the cohort study and were called at random. Four focus groups (two for registered and two for non-registered FSWs) were conducted with six FSWs in each focus group. The focus groups founded that nine out of 11 (81.8%) non-registered noted that they did not want their profession to be discovered by their loved ones. The findings from the focus groups regarding the favoured interventions to improve the current registration policy among non-registered FSWs along with the methods used and sample size can be found in the supplementary files, S1 Table and S2 Table respectively.”

The included supplementary files are:

**S1 Table. Favoured interventions to improve the current registration policy among non-registered FSWs.**

|  | Group 1 | Group 2 |
| --- | --- | --- |
| 1 | Changing the design of the booklet (smaller/removing the word 'health booklet'/no writing on it apart from the name) | Access to mental health advice or support |
| 2 | Access to literacy, training or skills development for FSWs | Quality medical care (friendly health staff, good medical care) |
| 3 | Removal of the central police database | Integration of medical visits, i.e. they can make the visit on any day |

**S2 Table. Focus group methods and study sample sizes.**

| **Wave** | **Method** | **Registered FSW** | **Non-registered FSW** | **Total** |
| --- | --- | --- | --- | --- |
| Wave 2 (2017) | Quantitative survey | 255 | 257 | 512 |
| Wave 3 (2020) | Focus groups | 2 | 2 | 4 |
| Wave 3 (2020) | Discrete choice experiment | 241 | 273 | 514 |

• 1st paragraph of pg. 8, there are again some issues with readability

We have rewritten this section and we hope that this is now clearer.

“Experimental design

The five chosen attributes and their corresponding levels (shown in Fig 1) have a possible 4^2 2^3=48 different combinations of outcome scenarios (two attributes with four levels and three attributes with two levels). All potential scenarios were not presented to each respondent due to likely respondent fatigue and low response rates. Using Ngene 1.2 software [13], a fractional factorial experimental design was used to reduce the number of scenarios whilst maximising the variation in the data. An efficient design was used, allowing for attributes to be independently varied over scenarios while minimising the standard errors of the parameter estimates. Specifically, we used a D-efficient design in which the D-error is minimised [13]. The final optimal design included 20 choice pairs. To reduce cognitive burden and fatigue for the respondents, these 20 choice pairs were ‘blocked’ and allocated across two versions of the DCE questionnaire each with ten choice pairs. All scenarios were not presented to each respondent due to likely respondent fatigue and low response rates. Using Ngene 1.2 software, a fractional factorial experimental design was used to reduce the number of scenarios whilst maximising the variation in the data. An efficient design was used, allowing for attributes to be independently varied over scenarios while minimising the standard errors of the parameter estimates. Specifically, we used a D-efficient design in which the D-error is minimised. The final optimal design included 20 choice pairs. To reduce cognitive burden and fatigue for the respondents, these 20 choice pairs were ‘blocked’ and allocated across two versions of the DCE questionnaire each with ten choice pairs. Next, we used Ngene to produce a fractional factorial experimental design that would reduce the number of scenarios presented to respondents whilst maximising the variation in the data. An efficient design was used, allowing for attributes to be independently varied over scenarios while minimising the standard errors of the parameter estimates. Specifically, we used a D-efficient design in which the D-error is minimised. The final optimal design included 20 choice pairs. To reduce cognitive burden and fatigue for the respondents, these 20 choice pairs were blocked and allocated across two versions of the DCE questionnaire each with ten choice pairs. Participants were randomly allocated to one of the two versions of the questionnaire.

Registered and non-registered FSWs received the same policy options to ensure the successful recommendation of an aligned registration policy which all FSWs. Each choice pair consisted of two alternative scenarios, labelled ‘Policy A’ and ‘Policy B’, and an option to choose neither (opt-out) for each of the ten choice pairs presented to them. For the ten choice pairs, each participant compared the two policy options ‘Policy A’ and ‘Policy B’ and had to choose their preferred policy option or whether to opt-out. They were told that if they chose neither they would prefer to remain in their current situation, either registered or non-registered FSW. Information regarding their current situation was gathered before the first task of the DCE. If the participant chose to opt out, we asked in a subsequent question to imagine they had to choose between ‘Policy A’ and ‘Policy B’ and we recorded their choice.

Allocation to one of the two versions of the DCE is stratified by registration status (i.e., registered and non-registered) and then randomly allocated within each group. The two blocks of policy scenarios were the same for both registered and non-registered FSWs. This was to ensure the successful recommendation of an aligned registration policy which satisfies both types of FSWs.

Participants were presented with the ten defined choice pairs consisting of two policies, labelled ‘Policy A’ and ‘Policy B’. The attributes and levels presented in the DCE were not discussed in-depth with the participants. Each policy choice pair was read out loud to the participant whilst showing the participant the associated diagram (see Fig 1, column 3 for the graphical representations). Negligible discussion defining each attribute was practiced by all enumerators to ensure the elicitation of truthful responses and minimise salience. The DCE manuscript can be found in the S1 File.

For each choice pair, participants were asked to choose their preferred policy out of the two policy options ‘Policy A’ and ‘Policy B’ presented, or if they preferred their current situation (e.g., current registration policy or to be non-registered) they could opt-out. Despite participants falling into two distinct categories, practicing legally or illegally, the baseline reference levels of the policy attributes of the DCE varied between participants within groups. Hence, the characteristics of the opt-out option varied between individuals. For registered FSWs, both misunderstanding and inconsistent application of the law played a huge role in their point of reference. For example, as previously discussed, where and how long a FSWs file is held often does not conform to regulation. To account for the misalignment of the baseline level of attributes, information regarding their current situation was gathered before the first task of the DCE (see Table 2). This information would allow for the appropriate analysis of the opt-out results. See Table 2 for participants baseline DCE attributes descriptive statistics. If a participant chose to opt-out, the next question forced the participant to make a choice between ‘Policy A’ and ‘Policy B’.

• You indicate that there were two phases of piloting. Is the first the completion of the DCE by 17 participants and the second the analysis to generate priors for the final experimental design? This was unclear.

Thank you for your comments. we are now making this clearer in the paper:

“Experimental design testing

The survey instrument was rigorously pretested at the design stage to verify the appropriateness of the precise wording and framing of the attributes and their corresponding levels. This included the testing of the pictures that illustrated each attribute and their corresponding levels as shown in column (3) of Fig 1. The DCE was piloted in two phases, initially to 10 participants. The first version was well understood by participants, so no changes were made. However, we piloted the DCE on an additional 7 participants in order to collect enough data to use as priors to generate the final efficient experimental design using Ngene 1.2 software [13]. Unexpected priors were highlighted during this stage and qualitative research was undertaken to shed light on them. For example, some participants who were not registered had a registration booklet. Participants were found to be sharing registration booklets and using out-of-date registration booklets to reduce the risk of facing imprisonment when working as a non-registered FSW.”

• Was randomization for the DCE stratified by registration status? It seems that way based on the results but it would be helpful to spell this out

Thank you for this comment – you are correct. We have made this clearer in the manuscript by including the following text:

“Allocation to one of the two versions of the DCE is stratified by registration status (i.e., registered and non-registered) and then randomly allocated within each group.”

RESULTS

• The table and the discussion of the results is very clear

Thank you.

DISCUSSION

• The discussion is completely lacking references and does not relate the findings of this study to any other literature, quantitative or qualitative.

Indeed, we have to point out that there is weak evidence on this topic given that a very few country globally have similar policy in place that are based on mandatory check up for FSWs. Senegal is currently the only country regulating sex work in Africa. As a result, it is not possible to link our results to similar contexts. Nonetheless we are not referring to two literature reviews showing that punitive laws for FSWs are counterproductive for HIV prevention.

“Whilst only a few countries have opted for compulsory medical check-ups to treat STIs among FSWs, two literature reviews found that punitive laws against FSWs are counterproductive to HIV prevention efforts[17, 18]. Our results give further evidence suggesting that laws could lead to an increase in stigma and human rights violations, and hence increasing the likelihood that, already vulnerable, FSWs practice sex work illegally. Urgent policy changes need to be considered by countries regulating sex workers using punitive laws to limit the negative effects of those policies on the mental and physical health of FSWs.”

• There are claims made, such as “In Senegal, psychologists are rare and their fees are not affordable…reinforcing the need for a state-funded service…” but these statements need corresponding citations.

We have now included the following references:

Ratele, Kopano. *The world looks like this from here: Thoughts on African psychology*. Wits university press, 2019.

Mayston, R., Frissa, S., Tekola, B., Hanlon, C., Prince, M., & Fekadu, A. (2020). Explanatory models of depression in sub-Saharan Africa: Synthesis of qualitative evidence. *Social science & medicine*, *246*, 112760.

Reviewer #2: This article presents the results of a discrete choice experiment among female sex workers (FSW) in Senegal about their preferences regarding the current registration policy in Senegal. The survey was embedded in a larger longitudinal survey among FSWs. Both registered and non-registered FSWs were surveyed.
The topic is relevant in terms of public health, and the survey results could be used to improve the current policy. However, the paper could benefit from several improvements and clarifications before publication.

The main issue is the way the article is organised. It seems that a previous version of the paper was not following the IMReD format, as suggested by the last paragraph of the introduction: “In Section 2, we present our methodology. Section 3 presents the data and descriptive statistics. Section 4 presents the method to overcome the selection bias associated with the decision to register along with the sensitivity analysis to test the conditional independence assumption. Results and a series of robustness checks are presented in Section 5 and discussed in Section 6. Finally, Section 7 concludes.” The paper has been reorganised to a more classical “Introduction – Method – Results – Discussion” plan, but incompletely, as suggested by this last paragraph of the introduction, not adequately updated.

Thank you for your comments, indeed we forgot to amend the last paragraph of the introduction, and this has now been amended. Here is the updated text:

“This study aimed to identify which aspects of the registration policy could be altered to make it more attractive to FSWs. The remainder of the paper is organized as follows. In Section 2, we present our methodology. Section 3 presents the results and section 4 discusses the findings. Finally, Section 5 concludes.”

Some methodological aspects and results are presented in the introduction that should be moved to the appropriate place. Other elements should be clarified (see the following comments). The authors could look at several recent papers from PLoS One as examples for better organising their contents.

We agree that our manuscript was not well structured. We have reorganised and hope this version is more readable.

I also have an issue with some elements of the conclusion, in particular, that sentence: “This could be addressed by making sure that all health centres are incorporating FSWs’ visits with the general population or with maternal care services.”

The conclusion drawn in the paper appears to be based on discussions held with local stakeholders or qualitative interviews conducted with health professionals, rather than solely on the presented results. However, these elements have not been explicitly presented in the methods or results sections of the paper. It is imperative that the conclusions of the paper are solely based on the presented results. To rectify this, the authors have two options: they may choose to remove this comment from the conclusion altogether or mention it in the discussion section as a potential solution that could be explored. Alternatively, the authors may choose to integrate the results of the workshop with stakeholders into the methods and results sections of the paper. This would ensure that the conclusions drawn are based on a solid foundation of empirical evidence.

We agree with your comments. The policy we suggest comes from the DCE results but indeed the acceptability of those policy changes were assessed using policy interviewers with relevant stakeholders. These results are presented in another paper that uses qualitative methods and that has been revised for PLOS One (Procureur et al, 2022). We are now referring to this paper in the following section:

“Focus groups with Female sex workers

In wave 3 of the data collection there were four focus groups, two with registered FSWs and two with non-registered FSW. Focus groups were conducted in a ‘non-directive’ approach, facilitated by a topic guide to understand their views on the registration policy and lasted on average 90 minutes. FSWs recruited for the focus group discussions had participated in at least one previous wave of the cohort study and were called at random. Four focus groups (two for registered and two for non-registered FSWs) were conducted with six FSWs in each focus group. The focus groups founded that nine out of 11 (81.8%) non-registered noted that they did not want their profession to be discovered by their loved ones. The findings from the focus groups regarding the favoured interventions to improve the current registration policy among non-registered FSWs along with the methods used and sample size can be found in the supplementary files, S1 Table and S2 Table respectively.”

INTRODUCTION
“In West Africa, the HIV epidemic is concentrated among FSWs, with 75% of HIV infections among men directly attributable to sexual intercourse with FSWs (Alary & Lowndes, 2004) (Alary M. , et al., 2013)” Do you have any more recent reference? Furthermore, this statement holds less validity for Senegal, where the epidemic is currently being driven by MSM.

This has been amended in the text:

“In West Africa, FSWs are one the key groups disproportionally affected with HIV, with 75% of HIV infections among men directly attributable to sexual intercourse with FSWs [2, 3]. In Senegal, the prevalence of HIV is 6.6% and FSWs are up to 9 times more likely to be infected with HIV than the general population [4].”

The introduction section of the current paper is recommended to be enhanced by incorporating additional references as the existing count of 14 references appears to be insufficient.

Furthermore, the inclusion of more recent references is also suggested as only two references have been published within the last five years.

We have increased the number of references. There are now 18 references included.

The introduction should first explore West Africa before being more focused on Senegal.

We now mention the situation in West Africa before focusing on Senegal.

Please provide more context and details about the current policy in terms of FSWs registration, and the current standard of care. About available services? It seems that half of participants already receive psychological support for example.

You are completely correct. We indeed realised in this study that some FSWs received some counselling from sex workers’ leaders or from hospital staff. However, note that this is informal support since the persons delivering this counselling have not been trained and the support is not provided in a transparent way, in fact many FSWs who may need this support may not have the relevant connection to get this support. We have now added this information in the descriptive statistics paragraph:

“In addition, we found that half of the participants benefited from some form of psychosocial support. However, from further investigation it was noted that this support was informally delivered by untrained peer sex workers and hospital staff which is likely to have hampered the transparency and efficacy of such support.”

“registration is unattractive to FSWs, explaining that 80% of FSWs in Senegal (Foley, 2010) and 57% in the capital city” said twice

Thank you for this comment. This has been amended in the text.

“This study aimed to identify which aspects of the registration policy could be altered to make it more attractive to FSWs. To do this, a discrete choice experiment (DCE) was administered to 241 registered and 273 non-registered FSWs. Five key policy attributes (registration file, registration identification, health centre confidentiality, health visit costs, psychological support) presented in the DCE were identified through previous research on the registration policy in Senegal and from a qualitative research with registered and non-registered FSWs (Procureur et al, 2022). Conditional logit models were conducted to analyse the DCE data. Results show that registration policy preferences of registered and non-registered FSWs were aligned. According to FSWs, ensuring confidentiality at health centre, replacing the physical registration proof by a QR code, providing psychological support, removing the registration file held by the police and only holding the registration file at the health centre whilst active in sex work would significantly improve the registration policy. As a result, while complete removal of police files and suppression of files held at the health centre when FSWs are no longer active would make registration much more attractive to FSWs, such amendments were deemed unfeasible given the current political context. Nevertheless, other changes potentially effective to increase registration could be introduced without the need to amend the law, including improving FSWs’ confidentiality, providing a psychosocial support service, improving the relationship of FSWs with the police and replacing the current registration card by a QR code to reduce stigma. “ This paragraph presents methods and results and should not be part of the introduction. The different elements should be presented later, at a more appropriate place.

We agree with your comments. We have removed this from the introduction and introduced a new paragraph in the methods which reads:

“A Discrete Choice Experiment (DCE) was implemented which allowed us to: (1) estimate the elements of a policy that are attractive or unattractive to FSWs, and (2) predict the registration uptake for the policy that combines the most attractive components. To do this, participants were required to make choices between a series of hypothetical, experimentally designed, but realistic registration policies, differing the characteristics of the registration policy. The DCE enabled the identification of policy attributes both deterring FSWs from registering and those that may persuade them to register.”

METHODS

“The DCE was embedded within the third wave of data collection of a longitudinal survey” Please provide more information about this longitudinal survey. Does the survey has a name? Has a protocol been published? Other analysis?

We are now providing the following additional evidence about the longitudinal study:

“Data collection and ethics

The DCE was embedded within the third wave of data collection of a longitudinal cohort study of 600 FSWs in Dakar, Senegal, noted earlier, and was carried out from 29 June to 28 July 2020. Data was collected face-to-face by trained enumerators in private rooms located near four of out five of the STI health centres in Dakar, Senegal (Pikine, Rufisque, Mbao, and Sebikotane). These STI health centres are now referred to as the study sites.

The cohort study follows FSWs in Dakar, Senegal and it was conceived to document and understand the socio-economic and behavioural determinants of risky sexual behaviours among FSWs. The cohort study began in 2015 (wave 1) and wave 2 was conducted in 2017. The aim of the third wave of data collection in 2020 was to define a policy change to address the main barriers of registration, increase the benefits of registration, provide evidence on the feasibility and to evaluate the effectiveness and cost-effectiveness of this policy change. Each wave of data collection for the cohort study received appropriate individual ethical clearance. Wave 3 received approval from the National Ethics Committee in Senegal (SEN19/88 n00037MSAS/DGS/DLM/DLSI) and the University College London ethics committee (17341/001).”

The study protocol has not been published but there have however been several papers published that use data from this study:

**Aurélia Lépine,** Carole Treibich and Ben d'Exelle. 2020. “[Nothing but the truth: Consistency and efficiency of the list experiment method for the measurement of sensitive health behaviours](https://www.google.com/url?q=https%3A%2F%2Fwww.sciencedirect.com%2Fscience%2Farticle%2Fpii%2FS0277953620305451&sa=D&sntz=1&usg=AOvVaw19BOTDwit4bQgrw2L3BOVz)”, Social Science & Medicine. 266(113326).

**Aurélia Lépine** and Carole Treibich. 2020. “[Risk preferences and HIV/AIDS: evidence from Senegalese sex workers](https://www.google.com/url?q=https%3A%2F%2Fwww.sciencedirect.com%2Fscience%2Farticle%2Fpii%2FS0277953620302392&sa=D&sntz=1&usg=AOvVaw2V_wBrqK48wAlT5qxROnKv)”, Social Science & Medicine. 256(113020).

Aurélia Lépine, Carole Treibich, Khady Gueye, Cheikh Tidiane Ndour, Peter Vickerman. 2020.“Estimating over-reporting in condom use among sex workers: Evidence from the list randomisation and the polling box methods”. Health Policy & Planning.

Carole Treibich and **Aurélia Lépine**. 2018. “[Estimating misreporting in sensitive health behaviours: Evidence from condom use of female sex workers in Senegal](https://www.google.com/url?q=https%3A%2F%2Fwww.researchgate.net%2Fpublication%2F327930555_Estimating_misreporting_in_condom_use_and_its_determinants_among_sex_workers_Evidence_from_the_list_randomisation_method&sa=D&sntz=1&usg=AOvVaw2QHaj5WPWTqvkUpEnj2321)”, Health Economics, 28(1), 144-160.

Seiro Ito, **Aurélia Lépine**, and Carole Treibich. 2018. “[The impact of becoming a registered female sex worker in Senegal on health and wellbeing”](https://www.google.com/url?q=https%3A%2F%2Fonlinelibrary.wiley.com%2Fdoi%2Ffull%2F10.1002%2Fhec.3791&sa=D&sntz=1&usg=AOvVaw33wPlPXzkVpIGf8yPB2NbJ), Health Economics, 27(11), 1627-1652.

Sara Haire, Aurélia Lépine, Carole Treibich, Daniel Effron. 2023 Can a Self-Affirmation Manipulation Encourage Female Sex Workers to Increase Their Uptake of HIV/STI Prevention Measures? Evidence from Senegal. *AIDS and Behaviour*, 1-14

Ethics: was it a specific approval for the DCE or a global approval for the longitudinal survey? When was approval obtained? Is there any reference (e.g. registration number) from the local ethic committee?

The DCE was one of the research objectives for the third wave and received approval part of this wave.

Each wave of the study received ethical clearance from a UK-based institution (LSHTM for wave 1 and wave 2, UCL for wave 3) and approval from the National Ethics Committee in Senegal.

We have now made this clear in the paper:

“Each wave of data collection for the cohort study received appropriate individual ethical clearance. Wave 3 received approval from the National Ethics Committee in Senegal (SEN19/88 n^0^0037MSAS/DGS/DLM/DLSI) and the University College London ethics committee (17341/001).”

Text and figure 1 should be harmonised (e.g. order and name of the attributes). Indicate on figure 1 what is the current standard of care. Is there a difference between a card and a booklet?

We apologise for not harmonising the text and the figure. This has now been updated in the text. Regarding your comment on the inclusion of the current standard of care, we cannot define a current standard of case given the heterogeneity in perceptions and experiences of FSWs regarding the current law. The reasons that the baselines may differ is due to lack of consistency in the application of the law. We have also changed card to booklet to match the French translation of “carnet sanitaire”. We have aligned all text to refer to the carnet sanitaire as a booklet as we agree that the utilisation of both words is confusing for the reader.

How was the confidentiality at the health centre presented to the participants?

Thank you for this question. The attributes were not discussed in-depth with participants, the attribute were just read out loud and we would show the associated diagram. We do so to minimise salience get more truthful answers from participants. However, in focus groups it was clear that confidentiality at the hospital referred to not having the sex workers status disclosed at the hospital.

We have included the following text to make this clearer:

“The attributes and levels presented in the DCE were not discussed in-depth with the participants. Each policy choice pair was read out loud to the participant whilst showing the participant the associated diagram (see Fig 1, column 3 for the graphical representations). Negligible discussion defining each attribute was practiced by all enumerators to ensure the elicitation of truthful responses and minimise salience. The DCE manuscript can be found in the S1 File.”

How was the question formulated? Is it about perception? What was the exact wording used in the questionnaires?

The confidentiality attribute in the DCE captures any perceived or real risks of having the sex worker status disclosed while at the hospital, either because the hospital staff reveals that the patient is a FSW or if the services for FSWs are not integrated and that the FSWs meets someone she knows while queuing or receiving the medical services.

We are now adding this information when presenting the confidentiality attribute in the section **DCE attributes and levels:**

“This attribute captures any perceived or real risks of having the sex worker status disclosed whilst at the hospital, either because the hospital staff reveals that the patient is a FSW or if the services for FSWs are not integrated and the FSWs meets someone she knows while queuing or receiving the medical services.”

In addition, we have now included the DCE manuscript as S1 File.

“Sex work is legal and regulated in Senegal. To practise it legally, you must:

- register with the authorities

- undergo routine monthly medical check-ups and test negative for STIs, at a monthly cost of around FCFA 2,000

- carry an up-to-date health booklet, confirming your compliance with routine medical check-ups

Your ID and photo will also be kept at the hospital and in a police file to prove your registration. These records will not be deleted if you leave sex work.

Being registered will not affect your human rights, you and your family members will still be able to travel outside Senegal, have access to the judicial system or the profession of your choice.

Senegalese law stipulates that unregistered sex workers risk a fine of 30,000 FCFA and a prison sentence of up to 6 months if arrested by the police.

We will now describe the different variants of the registration policy. There are ten questions, and each question presents two policy options that we would like you to compare. We'll ask you to tell us whether you prefer policy A, or policy B or neither.

Each policy option described depends on the following elements: file, proof of registration, confidentiality at the hospital, free medical visits, and psychosocial follow-up.

Remember, there are no right or wrong answers.

Once the task has been explained, the enumerator shows the participant the policy options. Whilst showing the participant the policy options on the screen, the enumerator asks:

- Imagine two registration policies: Policy A and policy B. Which do you prefer?

The responses to the first question are Policy A, Policy B or neither (opt-out) e.g., if unregistered, remain unregistered or if registered, they prefer the current policy. If the participant choses to opt-out, a question that forces a choice between policy A or B follows:

- For registered FSWs: You prefer the current policy, but which of policy A or B do you prefer?
- For unregistered FSWs: You prefer to remain unregistered, but which of policy A or B do you prefer?”

What is Ngene? A software? Please be explicit and add a reference.

Ngene 1.2 is a software which generates experimental designs used in stated choice experiments, to produce a fractional factorial experimental design that would reduce the number of scenarios presented to respondents whilst maximising the variation in the data. We are now adding this information and the relevant reference.

“Ngene 1.2 software [13], a fractional factorial experimental design was used to reduce the number of scenarios whilst maximising the variation in the data. An efficient design was used, allowing for attributes to be independently varied over scenarios while minimising the standard errors of the parameter estimates. Specifically, we used a D-efficient design in which the D-error is minimised [13].” <https://choice-metrics.com/NgeneManual120.pdf>

used
“Specifically, we used a D-efficient design in which the D-error is minimised.” Please explain. Any reference?

<https://link.springer.com/chapter/10.1007/978-3-030-62669-3_3>

<https://choice-metrics.com/NgeneManual120.pdf>

We have now added this paragraph to make this clearer:

“The five chosen attributes and their corresponding levels (shown in Fig 1) have a possible $4^{2}2^{3}=48$ different combinations of outcome scenarios (two attributes with four levels and three attributes with two levels). All potential scenarios were not presented to each respondent due to likely respondent fatigue and low response rates. Using Ngene 1.2 software [13], a fractional factorial experimental design was used to reduce the number of scenarios whilst maximising the variation in the data. An efficient design was used, allowing for attributes to be independently varied over scenarios while minimising the standard errors of the parameter estimates. Specifically, we used a D-efficient design in which the D-error is minimised [13]. The final optimal design included 20 choice pairs. To reduce cognitive burden and fatigue for the respondents, these 20 choice pairs were ‘blocked’ and allocated across two versions of the DCE questionnaire each with ten choice pairs.

Allocation to one of the two versions of the DCE is stratified by registration status (i.e., registered and non-registered) and then randomly allocated within each group. The two blocks of policy scenarios were the same for both registered and non-registered FSWs. This was to ensure the successful recommendation of an aligned registration policy which satisfies both types of FSWs. “

“We analysed the data and used the regression coefficients from the analysis of the pilot as priors to generate the final efficient experimental design.” Unclear for me. Could you explain? Clarify?

We are not sure if you are familiar with the DCE design but given the number of attributes and options selected we could have asked a combination of 48 questions to every participant. However, we know that some combinations are dominated and will not be chosen so we used the data from the pilot to reduce the number of questions asked in the DCE to make it more efficient.

In terms of data policy, it seems that the data are not available on a data repository.

We will be adding the data on the repository and have uploaded the data for replication purpose.

RESULTS

Table 1: please provide an informative title. Maybe not a good idea to mix some DCE results with a description of study participants.

I have divided table 1 into two tables and have renamed them.

**Table 1. Respondent demographic and sex work characteristics.**

| **Variables** | **Registered FSWs** | | **Non-registered FSWs** | |
| --- | --- | --- | --- | --- |
|  | **N** | **Mean/No.** | **N** | **Mean/No.** |
| ***Socio-demographic characteristics*** |  |  |  |  |
| Age, year (IQR) | 241 | 39.44 (32 – 47) | 273 | 38.60 (30 – 47) |
| Divorced or separated (%) | 241 | 181 (75.1) | 273 | 181 (66.30) |
| Working in sex work, months (IQR) | 241 | 99.48 (48 - 120) | 273 | 84.28 (36 - 120) |
| Went to school (%) | 241 | 117 (48.55) | 273 | 147 (53.85) |
| Household size (IQR) | 241 | 5.36 (1 - 7) | 273 | 8.33 (5 - 11) |
| Risk preferences in general (1 to 10) (IQR) | 241 | 3.89 (2 - 5) | 273 | 4.08 (3 - 5) |
| Risk preferences in sex (1 to 10) (IQR) | 241 | 1.96 (0 - 3) | 273 | 2.19 (0 - 3) |
| Life satisfaction (1 to 5) IQR) | 241 | 3.07 (2 - 4) | 273 | 3.24 (3 - 4) |
| ***Sex work activity*** |  |  |  |  |
| Monthly sex work income (CFAF) | 238 | 159,987.40 (60,000 - 200,000) | 273 | 101,754.60 (50,000 - 1,200,000) |
| Work mostly in bars or brothels (%) | 241 | 84 (34.85) | 273 | 32 (11.72) |
| Work mostly at home (%) | 241 | 67 (27.80) | 273 | 109 (39.93) |
| Has only occasional clients (%) | 241 | 11 (4.56) | 273 | 7 (2.56) |
| Has only regular clients (%) | 241 | 74 (30.71) | 273 | 266 (97.44) |
| Last client was a regular client (%) | 241 | 170 (70.54) | 273 | 245 (89.74) |
| Declared use of condom with last client (%) | 241 | 235 (97.51) | 273 | 263 (96.34) |
| Number of clients within a week (IQR) | 241 | 6.30 (0 - 9) | 273 | 4.79 (0 - 6) |
| Price of last sex act (CFAF) | 241 | 153,44.40 (5,000 - 18,000) | 273 | 14,909.96 (5,000 - 20,000) |
| ***Link with the authorities and the health system*** | |  |  |  |
| Police violence in the last 12 months (%) | 241 | 20 (8.30) | 273 | 10 (3.66) |
| Notes: N stands for the number of observations; CFAF: CFA francs | | | | |

**Table 2. Participant baseline levels of DCE attributes and opt-out statistics.**

|  | **Registered FSWs** | | **Non-registered FSWs** | |
| --- | --- | --- | --- | --- |
|  | **N** | **Mean/No.** | **N** | **Mean/No.** |
| **DCE baseline levels for participants** |  |  |  |  |
| File |  |  |  |  |
| No file (%) | 241 | 14 (5.81) | 271 | 265 (97.79) |
| File at health centre (%) | 241 | 149 (61.83) | 271 | 5 (1.85) |
| File at health centre and police (%) | 241 | 78 (32.37) | 271 | 1 (0.37) |
| Length of time of file |  |  |  |  |
| *File with health centre* |  |  |  |  |
| File at health centre whilst FSW (%) | 142 | 136 (95.77) | 3 | 3 (100.00) |
| File at health centre for life (%) | 142 | 6 (4.23) | 3 | 0 (0.00) |
| *File with police* |  |  |  |  |
| File with police whilst FSW (%) | 74 | 68 (91.89) | 1 | 0 (0.00) |
| File with police for life (%) | 74 | 6 (8.11) | 1 | 1 (100.00) |
| Does not have a ‘carte sanitaire’ (%) | 241 | 9 (3.73) | 272 | 263 (96.69) |
| Believe that health centres are confidential (%) | 240 | 235 (97.92) | 248 | 209 (84.27) |
| Attends health centre visits (%) | 241 | 237 (98.34) | 272 | 205 (75.37) |
| Receives free care during health centre visits (%) | 236 | 101 (42.80) | 204 | 141 (69.12) |
| Receives psychological support (%) | 241 | 146 (60.58) | 269 | 108 (40.15) |
| **DCE opt-out** |  |  |  |  |
| Opt-out at least once (%) | 241 | 21 (8.71) | 273 | 43 (15.75) |
| Opt-out rate of all choices (%) | 2410 | 32 (1.33) | 2730 | 233 (8.53) |
| Notes: N stands for the number of observations. FSW: Female sex worker. | | | | |

What is Opt-Out in this table? A result of the DCE?

We have now specified this.

“The two opt-out statistics presented in Table 2 are (1) the percentage of participants that opted-out of making a choice between Policy A and Policy B at least once and (2) the percentage of participants that opted-out for each one of the 10 policy scenarios presented. 43 non-registered FSWs out of 273 (15.75%) and 21 registered FSWs out of 241 (8.71%) chose the opt-out option at least once when presented with the 10 policy scenarios. The opt-out rate of all choices (both registered and non-registered FSWs) was very low 5.16% (265 out of 5140 choices), 1.33% (32 out of 2410 choices) for registered FSWs and 8.53% (233 out of 2730 choices) for non-registered FSWs.”

What is the item “File”? Current situation of the participant? What is “Length of time of file”? Current situation? If so, how do they know that the file will be there for life or just whilst FSW?

Thank you for your comment. We have now better explained each attribute and now make clear that the current situation of the participant is her perception of her situation for each attribute. This depends on the belief that the participant has on the way the policy is implemented.

The following text regarding the file has been amended:

“The first attribute shown in Fig 1 is the location, or with which authority, a FSWs file is held and for how long their file remains active. According to the law, at the time of registration at the health centre a file is created. This file is then shared with the police. The lawful process for when a FSW decides to leave sex work is that she should notify the health centre and then both the health centre and the police should destroy her file. However, in practice, the location of where and the length of time that the file is held for is often not in keeping with regulation. Files collated at the health centre are not always shared with the police and because of the perceived likelihood that they will return to sex work, health centres do not consistently destroy files when a FSW notifies them that they have left sex work. Discussions with FSW group leaders and FSWs highlighted that having sex work files held by police for life may cause difficulties due to stigma by the police.”

The following text explains the reason we use the “current situation”:

“Participants were presented with the ten defined choice pairs consisting of two policies, labelled ‘Policy A’ and ‘Policy B’. Participants were asked to choose their preferred policy out of ‘Policy A’ and ‘Policy B’ presented, or if they preferred their current situation (e.g., current registration policy or to be non-registered) they could opt-out. Despite participants falling into two distinct categories, practicing legally or illegally, the baseline reference levels of the policy attributes of the DCE varied between participants within groups. Hence, the characteristics of the opt-out option varied between individuals. For registered FSWs, both misunderstanding and inconsistent application of the law played a huge role in their point of reference. For example, as previously discussed, where and how long a FSWs file is held often does not conform to regulation. To account for the misalignment of the baseline level of attributes, information regarding their current situation was gathered before the first task of the DCE (see Table 2). This information would allow for the appropriate analysis of the opt-out results. See Table 2 for participants baseline DCE attributes descriptive statistics. If a participant chose to opt-out, the next question forced the participant to make a choice between ‘Policy A’ and ‘Policy B’”

Average marginal effects should be explained in the methods, including the note of Table 2. It seems that they are more specifically marginal contrasts, i.e. the difference between marginal predictions. Be aware that there is currently no consensus around the wording “marginal effects” and that it could refer to many things, see for example [https://www.andrewheiss.com/blog/2022/05/20/marginalia/](https://eur01.safelinks.protection.outlook.com/?url=https%3A%2F%2Fwww.andrewheiss.com%2Fblog%2F2022%2F05%2F20%2Fmarginalia%2F&data=05%7C01%7Cs.szawlowski%40ucl.ac.uk%7Cd79e90fe4f444dc3b1f508db44dfc7dc%7C1faf88fea9984c5b93c9210a11d9a5c2%7C0%7C0%7C638179500532714059%7CUnknown%7CTWFpbGZsb3d8eyJWIjoiMC4wLjAwMDAiLCJQIjoiV2luMzIiLCJBTiI6Ik1haWwiLCJXVCI6Mn0%3D%7C3000%7C%7C%7C&sdata=OxSUD4L2BDwr1vtgmvdkLJp14QqvRVWv1Jxiji7Vayg%3D&reserved=0)

Note that we are not predictions, so the coefficients are not marginal contrasts, they are average marginal effects, i.e., the average change in the probability to choose the policy when the attribute increases by one unit.

Table 2 requires a more comprehensive and descriptive title to enhance its readability.

Given that we have updated the tables, Table 2 is now Table 3. We have amended the title to read “Table 3. FSW policy preferences.**”.**

Additionally, the authors could consider presenting the marginal contrasts in percentage points (as in the text), which would facilitate the interpretation of the results by the readers.

We appreciate that would make sense in other context but in the context of a DCE, this would not be very informative given that the outcome of interest is a preference for a policy.

The use of standard errors in the table is not convincing as they are not easy to interpret. A more suitable option to display uncertainty would be to use 95% confidence intervals.

We have now reformatted the table to also show the 95% CI. See Table 3 in the manuscript.

**Table 3. FSW policy preferences.**

|  | **Average Marginal effects** | |
| --- | --- | --- |
|  | **Registered FSWs** | **Non-registered FSWs** |
| **File (ref = File at health centre and police for life)** |  |  |
| File at health centre whilst a FSW | 0.146*** | 0.170*** |
|  | [0.119 - 0.174] | [0.146 - 0.195] |
| File at health centre for life | 0.030*** | 0.014 |
|  | [0.011 - 0.050] | [-0.004 - 0.032] |
| File at health centre and police whilst a FSW | 0.082*** | 0.106*** |
|  | [0.055 - 0.109] | [0.082 - 0.129] |
| **Proof of registration (ref = Current booklet)** |  |  |
| Current booklet without the words 'carnet sanitaire' | 0.040*** | 0.033*** |
|  | [0.019 - 0.062] | [0.012 - 0.053] |
| Smaller version of the current booklet | 0.006 | 0.018 |
|  | [-0.016 - 0.028] | [-0.004 - 0.040] |
| QR code | 0.059*** | 0.035*** |
|  | [0.036 - 0.082] | [0.015 - 0.054] |
| **Confidentiality at the heath centre (ref = Confidentiality not guaranteed)** |  |  |
| Confidentiality guaranteed | 0.260*** | 0.221*** |
|  | [0.239 - 0.282] | [0.202 - 0.241] |
| **Cost of health centre visits and tests (ref = 2 000 FCFA)** |  |  |
| Free | -0.007 | 0.003 |
|  | [-0.020 - 0.005] | [-0.008 - 0.014] |
| **Psychological support (ref = No support)** |  |  |
| Psychological support | 0.051*** | 0.057*** |
|  | [0.031 - 0.072] | [0.039 - 0.075] |
| N | 4,794 | 5,340 |
| Clusters in ID | 241 | 270 |
| Prob > chi2 | 0 .0000 | 0 .0000 |
| Log pseudolikelihood | -1100.9688 | -1381.9941 |
| AIC | 2219.938 | 2781.988 |
| BIC | 2278.214 | 2841.235 |
| Coefficients are average marginal effects. ***p≤0.01, **p≤0.05, *p≤0.1 N stands for the number of observations. Standard errors are robust and clustered at the individual level. 95% confidence intervals in square brackets. The marginal effect is calculated by dividing the coefficient of the attribute by the change in the independent variable. The average marginal effects of each attribute were calculated to understand how much the rate of registration changes when an attribute changes. To do this you calculate the marginal effect for each individual i in the sample and then average the marginal effect over all sampled individuals. CFAF: CFA francs. FSW: Female sex worker. Reference categories are as follows: File – health and police file for life; Proof of registration – current booklet; Confidentiality at the health centre – confidentiality not guaranteed; Cost of medical visits and tests – paid (2 000 FCFA) and; Psychological support – no psychological support. | | |

To facilitate the reading of the results, the authors could create a new paragraph each time they present a new dimension.

In line with your comment, we have amended this in the manuscript and agree is it much easier for the reader to understand.

DISCUSSION

Are there similar surveys in the literature? Could the authors compare their results with other studies?

Unfortunately, there are no similar surveys in the literature given that this policy is only implemented in a very few countries globally (e.g. Senegal, Macedonia or some regions like Tijuana in Mexico). We have amended the first paragraph of the discussion to make this clear:

Our study explained why a large majority of FSWs in Senegal preferred to practice sex work illegally rather than register as a FSW.

Moreover, it identified policy changes that would make registration more attractive to FSWs.

This was done using a DCE to identify attributes of the registration policy that deterred FSWs from registering and those that encouraged them to register.

Overall, an important finding is that preferences of registered and non-registered FSWs were aligned, meaning that changes aiming at increasing registration would also improve the wellbeing of registered FSWs.

Please see the following updated text in the discussion:

“Senegal is the only African country where sex work is legalised and regulated, and this study is the first to use a DCE to elicit the registration policy preferences of FSWs in Africa. The findings identified registration policy attributes that both deterred FSWs from registering and those that encouraged them to register. Furthermore, to the best of our knowledge there are no other similar studies in non-African countries. While only a few countries globally have implemented legislation which requires FSWs to register and follow compulsory check-ups/ STI testing, our study sheds light on the issues that would arise with such laws. Our results also identified important design aspects to consider when implementing such a policy.”

Could results be replaced in context?

These results could be used to support other countries looking to implement a registration policy for sex workers. The following paragraph is included as the penultimate paragraph in the discussion (before the limitations):

“Whilst only a few countries have opted for compulsory medical check-ups to treat STIs among FSWs, two literature reviews found that punitive laws against FSWs are counterproductive to HIV prevention efforts [17, 18]. Our results give further evidence suggesting that laws could lead to an increase in stigma and human rights violations, and hence increasing the likelihood that, already vulnerable, FSWs practice sex work illegally. Urgent policy changes need to be considered by countries regulating sex workers using punitive laws to limit the negative effects of those policies on the mental and physical health of FSWs.

The findings of the study and proposed design of the registration policy focuses on improving FSWs confidentiality and mental health and combating the potential issues of punitive laws. These issues are universally a challenge for FSWs. Hence, if looking to implement laws to regulate sex work via compulsory health check-ups or STI testing, this study is likely to also support the design of an acceptable and feasible policy for sex workers in other settings.”
